# Improvement of memory and learning by intracerebroventricular microinjection of T3 in rat model of ischemic brain stroke mediated by upregulation of BDNF and GDNF in CA1 hippocampal region

**DOI:** 10.1186/s40199-017-0169-x

**Published:** 2017-02-15

**Authors:** Tahmineh Mokhtari, Mohammad Akbari, Fatemeh Malek, Iraj Ragerdi Kashani, Tayebeh Rastegar, Farshid Noorbakhsh, Mahmoud Ghazi-Khansari, Fatemeh Attari, Gholamreza Hassanzadeh

**Affiliations:** 1 Department of Anatomy, School of Medicine, Tehran University of Medical Sciences, Tehran, Iran; 2 Department of Immunology, School of Medicine, Tehran University of Medical Sciences, Tehran, Iran; 3 Department of Pharmacology, School of Medicine, Tehran University of Medical Sciences, Tehran, Iran; 4 Department of Neuroscience, School of Advanced Technologies in Medicine, Tehran University of Medical Sciences, Tehran, Iran

**Keywords:** Triiodothyronine, BDNF, GDNF, CA1, Stroke

## Abstract

**Background:**

Ischemic stroke is a common leading cause of death and disability with lack of effective therapies. In this study, T3 was intra-ventricularly injected to evaluate gene expression and protein concentration of and brain-derived neurotrophic factor (BDNF) and Glial cell-derived neurotrophic factor (GDNF) in hippocampal CA1 region in rat model of brain ischemia/reperfusion (I/R).

**Methods:**

In this study, transient middle cerebral artery occlusion (tMCAo) was used as model of ischemic brain stroke. Rats were randomly divided in four groups of Co, Sh, tMCAo and tMCAo + T3. Then, a single dose of intra-ventricular T3 was administered via a Hamilton syringe. Passive avoidance test was used as behavioral investigations. After 21 days, the animals were sacrificed and their brains were used for molecular and histopathological studies.

**Results:**

T3 significantly improved the learning and memory compared with tMCAo group according to Morris water maze findings *(P < 0.05)*. Step-through latency (STL) significantly decreased in tMCAo group *(P < 0.05).* There were significant increase in the STL of T3 group compared with tMCAo group *(P < 0.05)*.A significant reduction in BDNF mRNAs and protein levels were observed in the tMCAo compared with Co and Sh group *(P < 0.05)*. A significant increase of BDNF and GDNF mRNAs and proteins was recorded in tMCAo + T3 group compared with Co, Sh and tMCAO groups *(P < 0.05)*.

**Conclusions:**

The results of current study demonstrated that T3 had therapeutic effects on cerebral ischemic stroke by increasing the neurotrophic factors (BDNF, GDNF) in CA1 region of hippocampus.

**Graphical abstract:**

The effects of intracerebroventricular microinjection of T3on memory and learning in rat model of ischemic brain stroke.

## Background

Stroke is considered as one of the most leading cause of long-term disability and around the world [1]. Disruption of blood flow in the main brain blood supplying arteries such as middle cerebral artery (MCA) is an important reason for ischemic stroke by induction of hypoxia and glucose deprivation [2]. A complex series of biochemical and molecular mechanisms including excitotoxic glutamatergic signaling, outburst of reactive oxygen species (ROSs), over-production of inflammatory mediators induce the pathogenesis of cerebral ischemia by sudden death of a portion of neurons which leading wide range of neurological defects [3–5]. It was shown that among brain neurons, pyramidal neurons of hippocampal CA1 region are sensitive to ischemia condition [6]. These neurons play critical roles in learning and memory functions. Passive avoidance memory impairment following brain ischemia is associated with degeneration of pyramidal neurons [7].

The lack of successful therapies leads to high mortality and poor prognosis of patients with brain ischemia [8]. Although, the early thrombolytic therapies were suggested to restoring the blood flow, reperfusion itself exacerbate injury in the infarct core, leading condition known as cerebral ischemia/reperfusion (I/R) injury [9, 10]. Up to now, therapeutic agents with different mechanism of anti-apoptosis [11, 12], antioxidant [13], anti-inflammation [14] have been evaluated in animal models following the brain ischemia. Recent studies confirmed that thyroid hormones (THs) have broad neuroprotective effects on the nervous system [15].

THs (triiodothyronine [T3] and thyroxine [T4]) are essential for brain development and morphogenesis, as mental retardation can be observed in cases with congenital hypothyroidism [16, 17]. The anti-edema properties of THs in transient middle cerebral artery occlusion (tMCAo) model of ischemic brain have been confirmed [18]. T3 is less than T4 but more active form of THs than T4 [19]. Moreover, a specific transporter known as monocarboxylate 8 (MCT8) transfers T3 through blood brain barrier (BBB) [20]. The genomic actions of T3 are related to binding of this molecule to a specific receptor, known as thyroid hormone receptors (THRs). THRs are associated with the level of local expression of T3 and THRs by acting as hormone-inducible transcription factor [21]. The neuroprotective effects of non-genomic T3 are induced though activation of nitric oxide and vasodilation [22]. The neuroprotective benefits of THs established prior to its neurological insult. THs can control glutamate production, decrease oxidative stress and metabolic demands of neurons [23]. Also, the neuroprotective effects of T4 are associated with increasing the neurotrophic factors (NFs) such as brain driven neurotrophic factor (BDNF) and glial cell-derived neurotrophic factor (GDNF). Moreover, it is probably related to anti-apoptotic and anti-inflammatory mechanisms [24].

BDNF is one of the most critical growth factors with positive effects on the survival and maintenance of neuronal functions in the central nervous system. This factor can induce the differentiation of neurons via stimulating the receptor kinases [25]. Previously, it has been demonstrated that intravenous administration of BDNF reduces the infarct size following brain ischemia [26, 27]. GDNF, with trophic activities on dopaminergic neurons, has been shown to have neuroprotective impacts after ischemic brain injury [28].

As the strong neuroprotective property of T3 was discussed above, a single dose of T3 was selected and intraventricularly injected after 24 h of ischemia reperfusion as a therapeutic approach to attenuate exacerbation of transient middle cerebral artery occlusion (tMCAo) model of ischemia. Particularly, following endpoints were examined in this investigation: (1) behavioral alteration, (2) neurotrophic factors (BDNF, GDNF) expression and (3) dark neurons in histopathological studies (H&E and Nissl staining).

## Methods

### Animals

Eighty male Wistar rats (26–28 months classified as old) weighting 270–320 g were housed in a condition with standardized temperature, humidity and 12 h light/dark cycle and kept in the cages with free access to food and water. The animals were kept in animal room and cared in accordance with the guidelines of Tehran University of medical sciences on animal care and approved by Animal Ethics Committee of this university.

### Transient middle cerebral artery occlusion (tMCAo)

Focal cerebral ischemia was induced using in the left hemisphere tMCAo method. Anesthesia was induced with 5.0% isoflurane (Baxter International) and spontaneously inhaled with 1.0–2.0% isoflurane in air via a mask. During the procedure, the body core temperature was maintained at 37°C with a recirculation pad and K module and controlled via an intra-rectal temperature probes and blood flow was monitored by Laser Doppler flowmeter (Moor Instruments). A midline neck incision was applied during the operation time to expose the left common carotid artery (CCA). Then, CCA was dissected from surrounding fascia and adjacent vagus nerve to find its bifurcation. Then, the internal carotid artery (ICA) was carefully isolated to conduct the monofilament. An intraluminal 4–0 nylon monofilament (Doccol Co., USA) was used to occlude the medial carotid artery (MCA). This filament was inserted into the CCA and conducted into ICA to block the origin of the MCA. To apply the sham operation, the surgery was performed and the filament was inserted into the ICA and immediately withdrew.

### Animal groups and treatments

Rats were randomly allocated to the following groups:

- Control group (Co): The normal rats without any procedure and intracerebroventricular (ICV) injection of solvent (phosphate buffered saline [PBS] with maximum dose of 0.01% dimethyl sulfoxide [DMSO]) at the same time of tMCAo + T3 group.
- Sham group (Sh): Sham-operated rats with ICV injection of solvent at the same time of tMCAo + T3 with group
- tMCAo group: Rats were subjected to occlusion for 1 h followed by 24 h reperfusion and solvent at the same time of tMCAo + T3 with group
- tMCAo + T3 group: Rats were subjected to occlusion for 1 h and a single dose ICV of T3 (25 ug/kg body) was administered 24 after reperfusion [20]. T3 was dissolved in DMSO and diluted with PBS to reach maximum dose of 0.01% DMSO and a total volume of 5 ul was used for ICV injection. T3 or solvent was immediately injected using Hamilton syringe into the left cerebral ventricle (according to the following coordinates: bregma: AP = −0.9 mm, ML = -1.8 mm (midline), and DV = 3.5 mm deep from the dura) [29]. The same volume of solvent was used in the other groups.

### Body weight evaluation

The body weight of animals was investigated on initial day of study (X1) and 1st, 7th and 21st (Xi) days following the injection. Then, the percentage of body weight change (BWC) was calculated by following formula:

$$ \mathrm{B}\mathrm{W}\mathrm{C}\ \left(\%\right) = \left(\mathrm{X}1\hbox{-} \mathrm{Xi}\right)/\left(\mathrm{X}1+\mathrm{Xi}\right)\times 100 $$

On day 21, the animals were sacrificed and hippocampal CA1 region was harvested to study the gene expression (*n* = 4 rats in each group) and protein concentration (*n* = 4 rats in each group). Then, total brain was removed for histopathological studies (*n* = 4 rats in each group).

### Behavioral study

#### Morris water maze

Morris water maze tasks mainly include orientation navigation and space exploration trials. The water maze apparatus consisted of circular tank (diameter: 130 cm, depth: 60 cm) filled with opaque water (depth: 30 cm, temperature: 25 ± 3°C). The pool was divided into four sections and a camera attached to a computer was placed above the center of apparatus.

A safe platform was submerged in the pool approximately 2 cm below water surface, 30 cm from the wall of the pool in the center of the northeast quadrant. Each trial was videotaped and the animals’ movement tracked using a computer assisted tracking system. Testing began 21 days post injury and the rats were examined for 5 days with four trials for each session. Parameters for the test were time to find the platform, distance traveled to find the platform, time spent in platform quarter and distance traveled in platform quarter. The 5 sessions was a spatial probe trial in which the platform was removed and the rats were placed into the core of the pool and allowed to swim for 90 s. This test measures swim strategies and working (short-term, trial-to-trial) and reference (longer-term, day-to-day) memory.

### Step-through passive avoidance test

Passive avoidance learning test was used for evaluation of the learning and memory performance in the rats (*n* = 8 rats). The apparatus was a shuttle-box consists of two separate illuminated and dark chambers. These chambers were connected through a guillotine door. To deliver electric shocks, the base of the both chambers was made from still grill. The different phases of study were as below:

#### Step 1: adaptation and habitation

One day before the tMCAo induction, adaptation phase was performed for each rat. In a single trail, each rat was placed in the illuminated chamber and allowed to enter the dark chamber. One h later, rat was put into the illuminated chamber while its back was to the guillotine door. After 5 s of habituation, the guillotine door was simultaneously opened and rat was allowed entering the dark chamber. The latency time (LT1) to enter into the dark chamber was recorded. The rats with more than 100 s LT1 were excluded.

#### Step 2: training

One hour before the surgery, rat was placed into the illuminated chamber. After rat entered the dark chamber, the door was closed as soon as the rat stepped into the dark chamber and an electric foot shock (75 V, 0.2 mA, 50 Hz) was immediately delivered to the floor grids for 3 s. The latency time (LT2) was taken in this step (in second). Then, the rat was removed from the dark chamber and placed into the home cage. This procedure was repeated every 2 min for three times. If the rats did not enter the dark chamber, the latency time was recorded as 120 s.

#### Step 3: retention trial

In the acquisition trial, the time of retention latency (step-through latency [STL]) to enter the dark chamber was recorded in the same way without foot shock four times after 24 h of reperfusion: 1) STL1: Step-through latency 24 h after T3 administration,2) STL3: Step-through latency 3 days after solvent and T3 administration, 3) STL7: Step-through latency 3 days after solvent and T3 administration and 4) STL21: Step-through latency 21days after solvent and T3 administration (As the cut-off, the latency time was recorded up to a maximum of 300 s) and the longest STL was recorded.

### Total RNA extraction and quantitative real-time PCR

Twenty one day later, four rats in each group were used for gene expression of BDNF and GDNF. The brain rapidly removed under anesthesia and placed in ice-cold 0.9% saline. Then, the coronal sections (1mm) were prepared using a brain matrix. According to the stereo-allocation atlas, left CA1 hippocampal region of rat was quickly isolated under a dissecting microscope [30]. The expression of BDNF and GDNF genes in hippocampal CA1 region (*n* = 4 rats per group) was measured using quantitative real time PCR. In this way, total RNA was extracted by TriPure Isolation reagent (Takara Bio Inc., Otsu, Japan) according to manufacturer instruction. Then, cDNA was synthesized from 1μg total RNA using PrimeScript RT Reagent Kit (Takara Bio Inc., Otsu, Japan) according to manufacturer guidlines. The relative levels of BDNF and GDNF mRNAs were determined using ABI PRISM 7500 real-time PCR system (Roche Diagnostics, Germany). The primers were designed using Allele ID software (version 6). The primer sequences were as follow: BDNF, forward primer 5’-ATAATGTCTGACCCCAGTGCC-3’, and reverse primer 5’- CTGAGGGAACCCGGTCTCAT -3’, GDNF, forward primer: 5’- GCGCTGACCAGTGACTCCAA -3’, and reverse primer: 5’- GCGACCTTTCCCTCTGGAAT-3’, the b-actin gene, forward primer 5'- ACAACCTTCTTGCAGCTCCTC-3' and reverse: 5'- CTGACCCATACCCACCATCAC-3'(considered as the internal control standard). Quantitative real-time PCR was performed in a Cycler (Light Cycler 2.0, Roche) using SYBR Green (Takara Bio Inc., Otsu, Japan). The thermal cycling conditions were 95°C for 30 s as initial activation step. It was followed by 40 cycles of denaturation step including 95°C for 5 s which combined with annealing/extension step at 60 °C for 20 s. The threshold cycles (Ct), chosen from the linear range, was used for each sample and converted to a starting quantity.

### Concentration of BDNF and GDNF proteins in CA1 region

The protein concentrations of BDNF and GDNF in CA1 region of four rats (in each group) were evaluated using ABCAM ELISA kits and their manufacturer's guidelines. Hippocampal CA1 region was harvested as described above. Briefly, lysis buffer containing 137 mM NaCl, 20 mM Tris–HCl (pH 8.0), glycerol (10%), Igepal (1%), 0.5 mM sodium vanadate, 1 mM PMSF, 0.1 mM EDTA and 0.1 mM EGTA was used for homogenizing CA1 region of hippocampus (*n* = 4 rats per group). Then the homogenized tissue was centrifuged at 14,000 rpm at 4 °C for 3 min. The supernatant was diluted using sample buffer and incubated on 96-well flat-bottom plates. These plates were previously coated with anti-BDNF and anti-GDNF monoclonal antibodies. After blocking, plates were subsequently incubated with polyclonal anti-rabbit antibody for 2 h and horseradish peroxidase for 1 h. For calculating the concentration, the color reaction with tetramethyl benzidine was quantified in a plate reader at 450 nm.

### Histopathological study

Four rats in each group were selected for light microscopy study. Under anesthesia, the brains were perfused through a transcardial perfusion of 200 mL normal saline followed by 200 mL of 4% paraformaldehyde (PFA, sigma) in 0.1 M phosphate buffer (pre-fixation). The brains were removed and cut coronally into 3–5 mm-thick sections included the dorsal hippocampus. Then the sections were post-fixed in 10% formalin 72 h at 4°C. For light microscopy observation, the samples were embedded in paraffin and 5 μm coronal sections (one from each five sections) were prepared using a rotary microtome (Leica Biosystems, Milan, Italy). The tissue sections stained with Hematoxylin and eosin (H&E) and Nissl methods (0.5% cresyl violet). In this way, the sections were dehydrated through graded alcohols (70, 80, 90, and 100% × 2), mounted in Canada balsam, and then analyzed using a light field microscope (Olympus, CX31, Tokyo, Japan). The intact and ischemic cells (dark neurons) in CA1 field were counted in the × 400 images prepared using a camera connected to the microscope [31].

### Statistical analysis

Results are expressed as mean ± S.E.M. The data were analyzed statistically by *t*-test and one way analysis of variance (ANOVA) with Tukey's post hoc statistical tests. Non-parametric test (Behavioral results were analyzed statistically using Kruskal-Wallis Test (nonparametric ANOVA) and Dunn's Multiple Comparisons for post-test. The significant level was set at *P* < 0.05.

## Results

### Effects of T3 on the percentage of body weight change in rats with tMCAo

The effect of intraventricular injection of T3 on percentage of body weight change (BWC) was evaluated after transient focal cerebral ischemia/reperfusion model of rat on day 1, 7 and 21.

**Fig. 1:**
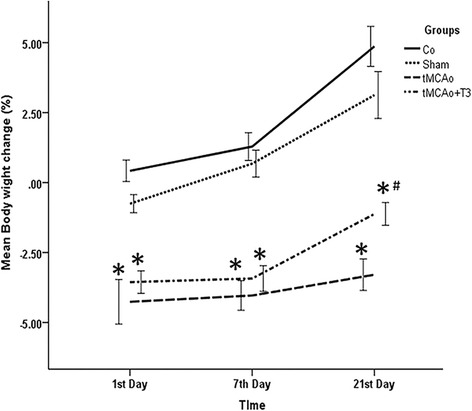
Effects of ICV injection of T3 on percentage of Body weight change following brain ischemia in rat on day 1, 7 and 21. *p < 0.05 compared to Co and Sh groups; ^#^ *p* < 0.05 compared to tMCAo group. Co: normal group with ICV injection of solvent, Sh: sham operated group with ICV injection of solvent; tMCAo: Ischemia induction group with ICV injection of solvent, tMCAo + T3: Ischemia induction group with ICV injection of T3

According to results, the mean BWC was significantly difference in the studied groups (*P < 0.05*, Fig. 1). Also, a significant decrease was recorded in the BWC of tMCAo and tMCAo + T3 groups compared with Co group on day 1, 7 and 21 after injection (*P < 0.05*, Fig. 1). In addition the BWC of tMCAo + T3 group significantly increased compared tMCAo group on day 21 after injection (*P < 0.05*, Fig. 1).

### Effects of ICV injection of T3 on learning and memory in rats with tMCAo

#### Results of Morris water maze

**Fig. 2:**
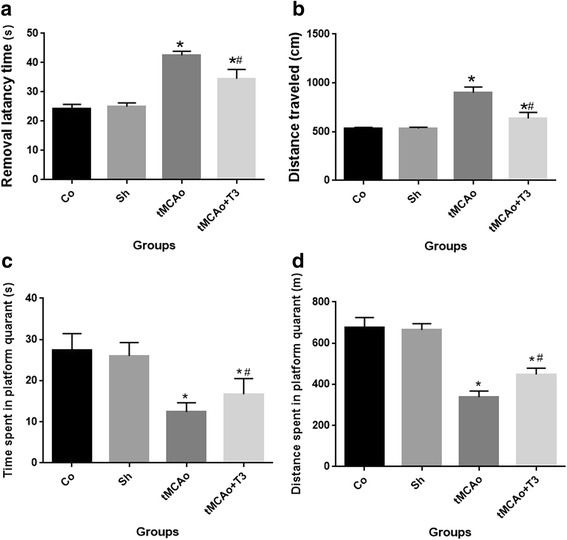
Effects of ICV injection of T3 on spatial memory and learning following brain ischemia in rat. **a** Mean latency to platform, **b** Mean Distance traveled to platform (**c**) time spent in the platform and (**d**) distance spent in the platform. * *p* < 0.05 compared to Co and Sh groups; #*p* < 0.05 compared to tMCAo group. Co: normal group with ICV injection of solvent, Sh: sham operated group with ICV injection of solvent; tMCAo: Ischemia induction group with ICV injection of solvent, tMCAo + T3: Ischemia induction group with ICV injection of T3

Treatments with T3 could improve spatial learning and memory in experimental group. According to Fig. 2, the mean latency (duration) time to reach the hidden platform (*P > 0.05*, Fig. 2a) and distance traveled in the Morris water maze (*P > 0.05*, Fig. 2b) were evaluated in tMCAo and tMCAo + T3 groups. However, significant decrease in the mean latency (duration) time to reach the hidden platform (*P > 0.05*, Fig. 2a) and distance traveled in Morris water maze (*P > 0.05*, Fig. 2b) was observed in T3 treated group compared to tMCAo group (*P > 0.05*, Fig. 2a and b).

Moreover, the probe trial revealed that tMCAo and drugs treated groups spent significantly less time (*P > 0.05*, Fig. 1c) and distances in the platform quadrant (*P* > 0.05, Fig. 2d) compared to control and sham groups (Co and Sh). Rats in tMCAo + T3 group spent significantly more time (*P > 0.05*, Fig. 2c) and distance in the platform quadrant (*P > 0.05*, 2d) compared to tMCAo group.

### Effects of ICV injection of T3 on STL in the passive avoidance test in rats with tMCAo

**Fig. 3:**
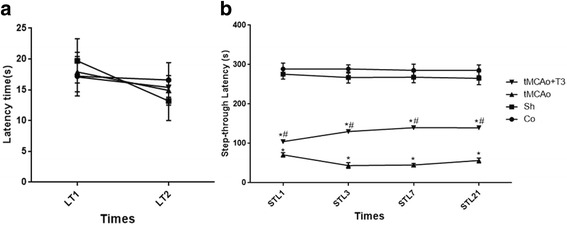
Effects of ICV injection of T3 on STL in the passive avoidance test following brain ischemia in rat. **a** LT1: Latency time at 24 h before to ischemia; LT2: Latency time at 1h before to ischemia; **b** STL1: Step-through latency 24 h after solvent and T3 administration; STL3: Step-through latency 3 days after solvent and T3 administration, STL7: Step-through latency 7 days after solvent and T3 administration, STL21: Step-through latency 21 days after solvent and T3 administration, *p < 0.05 compared to Co and Sh groups; #*p* < 0.05 compared to tMCAo group. Co: normal group with ICV injection of solvent, Sh: sham operated group with ICV injection of solvent; tMCAo: Ischemia induction group with ICV injection of solvent, tMCAo + T3: Ischemia induction group with ICV injection of T3

There was no significant difference in the LT1 and LT2 of different groups (Fig. 3a). There was significant difference in the STL of study groups at different evaluation times (*p < 0.05*, Fig. 3b). There was significant reduction in the STL of tMCAo group compared with Sh and Co groups on day 1, 3, 7 and 21 (*p < 0.05*, Fig. 3b). A significant decrease was observed in the STL of tMCAo + T3 group compared with Sh and Co groups on day1, 3, 7 and 21 (*p < 0.05*, Fig. 3b). Moreover, a significant increase was shown in the STL of group with T3 administration compared with tMCAo on day 1, 3, 7 and 21 (*p < 0.05*, Fig. 3b).

### Effects of T3 on BDNF and GDNF gene expression in hippocampal CA1 region in rats with tMCAo

**Fig. 4:**
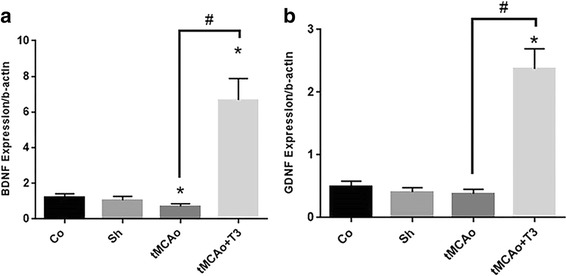
Effects of ICV injection of T3 on neurotrophic factor gene expression of hippocampal CA1 region following brain ischemia in rat. **a** BDNF gene expression, **b** GDNF gene expression. *p < 0.05 compared to Co and Sh groups; ^#^ *p* < 0.05 compared to tMCAo group. Co: normal group with ICV injection of solvent, Sh: sham operated group with ICV injection of solvent; tMCAo: Ischemia induction group with ICV injection of solvent, tMCAo + T3: Ischemia induction group with ICV injection of T3

According to the Fig. 3a, there were significant differences in the gene expression of BDNF and GDNF between study groups. A significant decrease was observed in the gene expression of BDNF in tMCAo group compared with Co and Sh groups (*p < 0.05,* Fig. 4a). There was significant increase in the gene expression of BDNF in tMCAo + T3 group compared with Co, Sh and tMCAo groups (*p < 0.05,* Fig. 4a). Furthermore, a significant increase was recorded in the gene expression of GDNF in tMCAo + T3 group compared with Co, Sh and tMCAo groups (*p < 0.05*, Fig. 4b).

### Effects of T3 on BDNF and GDNF protein concentration in hippocampal CA1 region in rats with tMCAo

**Fig. 5:**
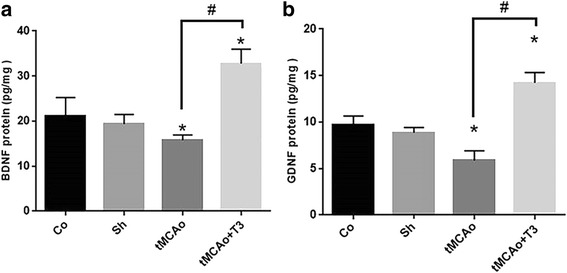
Effects of ICV injection of T3 on neurotrophic factor protein concentration of hippocampal CA1 rejoin following brain ischemia in rat. **a** BDNF and **b** GDNF proteins. *p < 0.05 compared to Co and Sh groups; ^#^ *p* < 0.05 compared to tMCAo group. Co: normal group with ICV injection of solvent, Sh: sham operated group with ICV injection of solvent; tMCAo: Ischemia induction group with ICV injection of solvent, tMCAo + T3: Ischemia induction group with ICV injection of T3

According to ELISA results, the protein concentration of BDNF was reduced in tMCAo group compared with Co and Sh groups (*p < 0.05*, Fig. 5a). There was significant increase in the concentration of BDNF protein in tMCAo + T3 group compared with Co, Sh and tMCAo groups (*p < 0.05*, Fig. 5a). The protein concentration of GDNF significantly was reduced in tMCAo group compared with Co and Sh groups (*p < 0.05*, Fig. 5b). Moreover, a significant increase was observed in the protein concentration of GDNF in tMCAo + T3 group compared with Co, Sh and tMCAo groups (*p < 0.05*, Fig. 5b).

### Effects of T3 on the number of dark neurons in hippocampal CA1 region of in rats with tMCAo

**Fig. 6:**
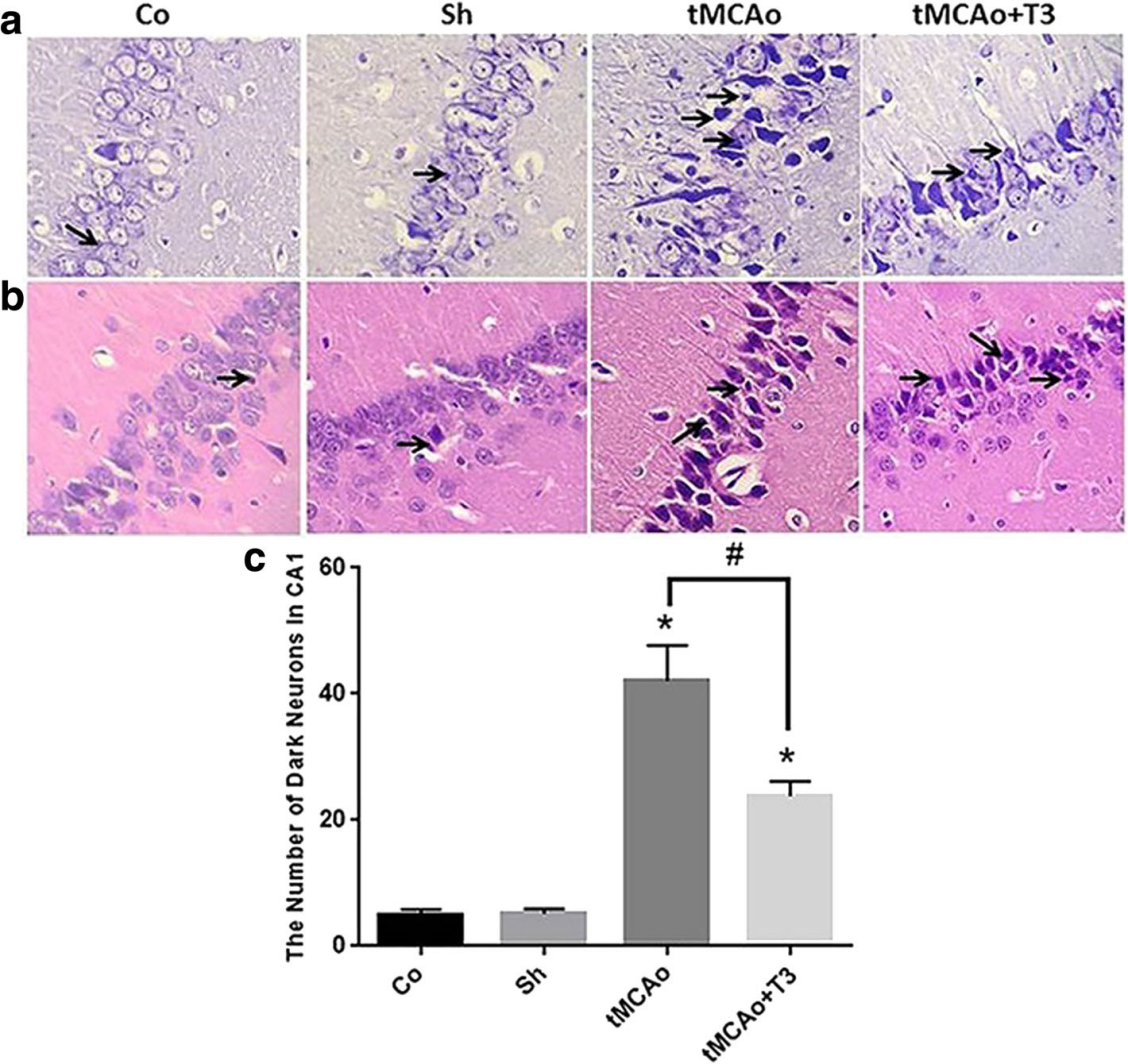
Effects of ICV injection of T3 on pyramidal neurons of CA1 region of hippocampus following brain ischemia in rat. **a** Nissl staining (arrows: dark neurons, ×400), **b** H&E staining (arrows: dark neurons, ×400), **c** Comparing the dark neuron number in different groups. *p < 0.05 compared to Co and Sh groups; ^#^ *p* < 0.05 compared to tMCAo group. Co: normal group with ICV injection of solvent, Sh: sham operated group with ICV injection of solvent; tMCAo: Ischemia induction group with ICV injection of solvent, tMCAo + T3: Ischemia induction group with ICV injection of T3

The number of dark neurons was calculated in CA1 region of hippocampus in the study groups. There was significant increase in the mean number of dark neurons in tMCAo and tMCAo groups compared with Co and Sh groups (*p < 0.05*, Fig. 6). A significant decrease was recorded in the mean number of dark neurons in tMCAo + T3 compared with tMCAo group (*p < 0.05*, Fig. 6).

## Discussion

In this study, tMCAo model was carried out for experimental evaluations. Different animal models were established to study the brain ischemia in the literature. Transients MCA occlusion (tMCAo) is a rodent model of ischemia that is widely used to analyze the mechanisms triggered by ischemic stroke and study the potential therapies [32, 33]. In brain ischemia, a cascade of pathological events and subsequent neuronal damages are induced within minutes of its onset [34]. These pathological events are associated with a complex process involving metabolic dysfunction, inflammation, neuronal necrosis and apoptosis, microvascular and endothelial dysfunction following an impaired blood flow [35]. Moreover, the neurons of some regions are susceptible to cerebral ischemia. The most common affected regions are cerebral cortex and striatum, while secondary cell death occurs in the CA1 region of hippocampus within 2–4 days after transient brain ischemia [36, 37]. These pathological events and following disabilities are confirmed in tMCAo model of brain ischemia stroke [38, 39].

The effects of different therapies with various mechanisms have been evaluated on the brain ischemia models to manage the pathogenesis and outcomes of this condition [11–14]. THs (T3 and T4) are essential for development, growth and metabolism especially in nervous system [19]. In addition, T3 plays important role in neurogenesis in the early stages of brain development [40, 41]. This active form of THs can bind to thyroid receptors (TRs) with much affinity than T4 which is more abundant [42]. In the current study, the effects of T3 (a single dose of 25ug/Kg) were evaluated on tMCAo model of ischemia in different aspects of behavioral, molecular and histopathological.

The finding of present study showed that the neurotrophins (BDNF and GDNF) expression pattern was disrupted in CA1 region of hippocampus due to tMCAo. This condition leads to sever deficits in expression of these neurotrophins. The similar result was demonstrated in recent study by Genovese et al. [24]. It was shown that the morphological changes in hippocampus were associated with regulation of mRNAs expression of neurotrophins [43]. Administration of neurotrophic factors following the brain ischemia was investigated in different studies. In a review article, Chen et al [23] showed that BDNF is a safe and potential agent with neuroprotective properties against ischemic brain injury. In addition, the neuroprotective effects of GDNF in the ischemic brain was evaluated by Duarte et al [28].

Different mechanisms have been suggested for beneficial effects of THs against neurological defects. The mechanism and molecular basis of THs against the toxicity conditions during the ischemia was investigated in different studies. Their ability to induce hypothermia, anti-edema, anti- apoptotic anti-inflammation and vasodilation activates are evaluated in different studies [15, 16, 22, 44]. THs regulate BDNF gene expression in different regions during the development [45–47] and maturation [48] of nervous system. Sui et al. [49] demonstrated that administration of THs increased BDNF gene expression in normal rat hippocampus by promoter-specific regulation of BDNF. Accordingly, Campolo et al [44] showed that post stroke ICV injection of T4 can increase BDNF and GDNF RNA transcripts and protein levels in hippocampal CA1 region. In a similar study, by Genovese et al. [24], T4 (1.1 mg/100 g BW) effects on ischemia model of stroke following reperfusion was evaluated. Their results revealed that T4 has therapeutic effects on brain ischemia through anti-apoptotic and anti-inflammatory mechanisms and regulation of NFs (BDNF and GDNF) expression in brain tissue in a rat model of acute ischemic stroke . It has been demonstrated that activation of NFs via THs is associated with their effects on transcriptional factors or epigenetic mediated processes (a mechanism for covalent modifications of the DNA or its related proteins without a change of DNA sequence) [50–52].

According to the results of current study, it was showed that the mean number of dark neurons was high in hippocampus CA1 region following cerebral ischemia. Moreover, the number of dark pyramidal neurons was reduced in T3 treated group in comparison with ischemic group. In a study by Rami and Krieglstein [53], it was shown that daily administration of T4 reduced hippocampal neuronal damage following brain ischemia. Their results indicated that neural density was increased 50% by T4 treatment compared with ischemic group [53]. Losi et al. [54] reported that rat hippocampal neurons were protected against glutamate toxicity by non-genomic T3 administration. Thus, both non-genomic and genomic mechanisms of T3 were involved in the protection of neurons and glial cells against the glutamate toxicity [44, 55, 56]. According to the results, the survival effects of T3 in decreasing the dark neurons may be associated with increasing NFs in CA1 region.

According to the Morris water maze and step-through finding, it was demonstrated that a single dose of T3 (ICV injection) improved memory and learning in treated group compared with ischemic group in short and long times. Low T3 concentration introduced as a strong predictor of stroke with a worse outcome according to significant human clinical evidence. In other study by Zhang et al. [57], it was suggested that low T3 level in the serum of patients with AIS was associated with unfavorable neurological outcomes. Previously, Hiroi et al. [22] evaluated the effects of T3 on transient cerebral ischemia in mice. They showed that a single bolus infusion of T3 rapidly increased the activation of PI3-kinase/Akt pathways activity in the brain, decreased cerebral infarct volume, and repaired neurological deficit score. In addition, they showed that T3 reduced blood pressure in focal cerebral ischemia via vasodilation mechanism related to eNOS contribution [22]. So, the administration of T3 prevented the worse outcomes of cerebral stroke in a schemial model of stroke. In addition, the ICV injection dose of 25ug/Kg was confirmed that did not induce thyro-toxicity in investigated animals in the present study. Drover et al. [58] recorded that injection of T3 (250 μg/kg dose for 14 consecutive days) was required after experimental hyperthyroidism induction in mice.

## Conclusion

According to these findings, it has been suggested that presence of T3 in brain play an essential role for modulation of NFs expression. The findings of current study confirmed the greater survival of CA1 hippocampal neurons in T3 treated animals via neurotrophic factors regulation and provide evidence to stimulate clinical development of T3 for use as effective therapy in ischemic brain stroke to reduce the cognitive impairment.

## Abbreviations

BDNF: Brain-derived neurotrophic factor
GDNF: Glial cell-derived neurotrophic factor
I/R: Ischemia/reperfusion
LT: Latency time
MCA: Middle cerebral artery
NFs: Neurotrophic factors
ROSs: Reactive oxygen species
STL: Step-through latency
T3: Triiodothyronine
T4: Thyroxine
THRs: Thyroid hormone receptors
THs: Thyroid hormones
tMCAo: Transient middle cerebral artery occlusion
